# Biofilm interfacial acidity evaluation by pH-Responsive luminescent nanoparticle films

**DOI:** 10.1016/j.bios.2020.112732

**Published:** 2020-10-22

**Authors:** Padryk Merkl, Marie-Stephanie Aschtgen, Birgitta Henriques-Normark, A. Sotiriou Georgios

**Affiliations:** a Department of Microbiology, Tumor and Cell Biology, Karolinska Institutet, SE-171 77, Stockholm, Sweden; b Department of Clinical Microbiology, Karolinska University Hospital, SE-171 76, Stockholm, Sweden; c Lee Kong Chian School of Medicine (LKC), Singapore Centre on Environmental Life Sciences Engineering (SCELSE), Nanyang Technological University, 639798, Singapore

**Keywords:** Calcium phosphate, Nanoparticle coating, Photoluminescence, pH sensor, Medical device, Nanophosphor

## Abstract

Biofilms are dense bacterial colonies that may adhere to the surfaces of medical devices and are major contributors to infections. These colonies are characterized by a self-produced matrix of extracellular polymeric substances (EPS). Bacterial biofilms are difficult to treat with the commonly used antibiotics partially because of their poor diffusion through the EPS and therefore require new targeted strategies to effectively fight them. Biofilms may produce an acidic microenvironment which can be exploited to design such targeted treatment strategies. However, there is currently a lack of high-throughput ways to determine the acidity of biofilms at their interface with the medical device. Here, a novel all-inorganic pH responsive system is developed from luminescent carbonated hydroxyapatite nanoparticles doped with Eu^3+^ ions which can determine the biofilm acidity fluorometrically due to carbonate removal in acidic environments that directly affects the nanoparticle luminescence. The pH responsive nanoparticles are in-situ deposited during their production onto substrates on which a variety of clinically-relevant biofilms are grown. The acidity of their interfacial (micro)environment depends on the bacterial species and strain even when differences in biofilm biomass are considered.

## 1 Introduction

Bacterial biofilms can form on both patient tissue and medical devices (such as titanium implants) and are the leading cause of persistent infections in patients (Römling et al., 2014; Römling and Balsalobre, 2012). These colonies of bacteria adherent to a surface are characterized by a dense self-produced matrix composed of extracellular polymeric substances (EPS). Biofilms show increased resistance to antibiotics and the immune system due to the diffusion-limited environment created by the EPS and the presence of dormant cells (Hall and Mah, 2017). Bacteria within a biofilm share genetic material more readily due to their close proximity and thus, antibiotic resistance rates are higher in bacterial biofilms than in planktonic bacteria (Flemming et al., 2016).

Therefore, there is a need to identify novel treatment strategies capable of killing established biofilms and/or prevent their formation on abiotic surfaces. Several such strategies, as recently reviewed by Koo et al. (2017) utilize stimulus-responsive materials that capitalize on pH changes inside the biofilm. For example, the medical device surface may be functionalized with a pH-triggered material allowing the release of an antimicrobial agent (Horev et al., 2015). Alternatively, pH dependent changes can also be exploited such as the acidic activation of free radical production by iron oxide (Fe_3_O_4_) nanoparticles that destroy the formed biofilm (Naha et al., 2019), or pH-induced aggregation of silver nano-particles to modulate their antimicrobial potency (Qiao et al., 2019). Thus, a deeper understanding of the acidic microenvironment formed by biofilms is crucial in the design and development of such intelligent solutions. The origin of biofilm acidification is attributed mainly to by-products of bacterial carbohydrate metabolism such as acetic and lactic acid, however, an additional contributing factor may be extra-cellular DNA found in the EPS (Schlafer et al., 2018; Wilton et al., 2016).

One common method for accurate pH determination involves the use of electrodes or microelectrodes (Miyanaga et al., 2007; Von Ohle et al., 2010; Wang et al., 2013; Xiao et al., 2013; Zaura et al., 2002). These have been applied to sense the pH of biofilms, however, such techniques require physical contact and insertion of a relatively bulky object within the biofilm which severely disturbs its structure and they cannot be applied to measurements of lateral spatial gradients. Biofilm pH has also been successfully measured using ratiometric fluorescent dyes that can show a change in their luminescence emission in low pH (micro)environments. However, such dyes suffer from poor photostability and therefore the data quality is liable to deteriorate over time (Bünzli, 2016). Some such pH sensors were developed as fluorescent surfaces (Albright et al., 2017; Gashti et al., 2016; Khoerunnisa et al., 2017), whereas others were with fluorescent dyes or particles labelled with dyes (Fulaz et al., 2019; Hidalgo et al., 2009; Schlafer et al., 2018; Schlafer and Dige, 2016; Vroom et al., 1999; Xiao et al., 2017) that aim to measure the pH throughout the entire biofilm and are therefore incubated with it for some time to allow their spatial distribution. These systems have been used to measure the highly inhomogeneous pH distribution found in biofilms, with gradients of more than 3 pH units measured between the biofilm surface and the interface on which the biofilm grows (Hidalgo et al., 2009). In order for the distributed pH sensors to provide spatial information on the pH distribution at particular locations within the biofilm they must be used in conjunction with confocal microscopy. This reliance on confocal microscopy results in time-consuming measurements requiring both highly specialized equipment and highly trained operators.

In this work, a novel all-inorganic pH sensor is developed based on luminescent nanoparticles of calcium phosphate (CaP) doped with Eu^3+^ ions (CaP:Eu^3+^) deposited as a film mimicking the biocompatible inorganic implant coatings that are often employed (Rao et al., 2014) to promote osseointegration (*e.g*. calcium phosphate, bioglass (Aldini et al., 2002; Nasiri et al., 2018)). These luminescent nanoparticle films are deposited on substrates during their gas-phase flame synthesis combining material production and device fabrication in a single-step and such substrates include titanium and plastics (Blattmann et al., 2015; Nasiri et al., 2016). The properties of the as-produced functional CaP:Eu^3+^ nanoparticle films are characterized in detail with emphasis on their photoluminescence and carbonate content, and applicability to a plate reader-friendly assay format. Their pH sensing properties are examined in various pH buffers to establish a structure-function relationship. The performance of the developed pH sensing nanoparticle films is demonstrated here by growing clinically-relevant biofilms on their surface and measuring the acidity of the interfacial biofilm-device microenvironment.

## 2 Materials and Methods

### 2.1 Nanoparticle film synthesis

CaP:Eu^3+^ nanoparticles were produced by flame spray pyrolysis (FSP) (Mädler et al., 2002). The liquid precursor solution was prepared by dissolving calcium acetate hydrate (99%, Sigma Aldrich) in a mixture (1:1 unless otherwise stated) of propionic acid (99.5%, Sigma Aldrich) and 2-ethylhexanoic acid (99%, Sigma Aldrich) under reflux at 70 °C for 1 h (Modified from Ataol et al. (2015)). Europium nitrate (99.9%, Alfa Aesar) was added to correspond to a 5 at% substitution of calcium and finally tributyl phosphate (99%, Sigma Aldrich) was added to yield a solution with a 0.4 M total inorganic concentration. The nominal ratio of calcium to phosphorous was varied between Ca/P 1.5 to Ca/P 2.19. The solution was fed through a capillary from a 50 mL syringe (SGE Analytical Science) by a syringe pump (New Era Pump Systems, Inc.) at a rate of 10 mL/min. The solution was dispersed by 5 L/min (EL-FLOW Select, Bronkhorst) of oxygen (>99.5%, Strandmöllen AB) with a pressure drop at the nozzle of 1.8 bar. The spray was ignited by a pre-mixed methane/oxygen (>99.5%, AGA Gas AB) flamelet with flow rates of 1.5 L/min and 3.2 L/min, respectively. Nanoparticles were collected on a glass fiber filter (Hahnemühle) further downstream with the aid of a vacuum pump (Mink, Busch). Deposition of CaP:Eu^3+^ films onto silicon chips (Micro-Tec P{100} diced, 5 mm squares 525 μm thick) was performed by positioning the flame directly below a water cooled substrate holder. The height of the substrate above the nozzle during deposition was 16 cm and the deposition time was 60 s. Following deposition, an in-situ annealing step was performed, in which a pure ethanol flame was pumped at a rate of 12 mL/min and dispersed by 3 L/min of oxygen, while the water cooling was turned off and the substrate was lowered to 11 cm above the nozzle. The annealing was performed for 20 s. Additional ex-situ annealing was performed using a CWF 13/5 furnace (Carbolite Gero).

### 2.2 Nanoparticle film characterization

The XRD diffractograms of the nanocoatings were collected using a Rigaku MiniFlex 600, CuKα radiation and analyzed with the PDXL2 software (Rigaku). The SEM micrographs were collected using a Gemini Ultra 55 (Zeiss) with secondary electron detector and 5 kV beam voltage or a Phenom Pharos (Thermofischer Scientific) with 5 kV beam voltage. Porosity of nanoparticle films was calculated from the well-established porosity of films made by flame aerosol deposition prior to annealing of 98% (Kemmler et al., 2013; Riefler and Mädler, 2010). The TEM images were collected using a Tecnai Spirit BioTWIN (FEI) with a 120 kV beam voltage. Fourier transform infrared spectroscopy with attenuated total reflection (FTIR-ATR) module was performed on the coatings with an Agilent Cary 630 instrument. The luminescence spectra were collected using an LS55 spectrometer (PerkinElmer) with plate reader attachment. The luminescence excitation and emission of the coatings was accomplished via fibre optics, both the excitation spot and emission collection area were larger than the CaP:Eu^3+^ films, allowing sampling of the complete film. The asymmetry ratio used is calculated as *A*_*R*_ = $\sum_{\text{n}=610\text{nm}}^{618\text{nm}}{\text{I}}_{\text{n}}/\sum_{\text{n}=586\text{nm}}^{594\text{nm}}{\text{I}}_{\text{n}}$ In from which the sensor response measured from the emission spectra of the CaP:Eu^3+^ coatings is defined as $S_{R}=1-A_{R}/A_{R}^{ini}$ where *A*_*R*_ is the measurement after treatment and $A_{R}^{ini\;}$ is the measurement of the dry film prior to treatment. Custom well plates (technical drawing in supporting information (SI)) were 3D printed (Wanhao duplicator 4 S) using a black ABS filament (PrimaValue). Various acetate buffers were prepared by mixing appropriate volumes of acetic acid and sodium hydroxide in ultrapure water and were verified with a pH meter (VWR pH 1100 L). Modified M9 minimal medium (preparation protocol in SI) at different pH values was prepared by adding acetic acid until the desired pH was achieved.

Quantification of calcium ion release from the CaP:Eu^3+^ films was performed after incubation of the films at 23 °C for 3 h. Measurements were performed both on the dissolved fraction in the supernatant incubated with the chips and on the undissolved fraction remaining on the chips after incubation. The undissolved fraction was dissolved for measurement by incubating the chips in Eppendorf tubes for 4 h in 30% acetic acid undergoing vigorous shaking. Volumes were adjusted to 4 mL for all samples by adding ultrapure water to allow for measurement using a Ca^2+^ ion selective electrode (PerfectION, Mettler Toledo) with an ion meter (SevenCompact, Mettler Toledo).

### 2.3 Bacterial growth

The bacteria were cultured on lysogeny agar plates from frozen stock. A single colony was then suspended in lysogeny broth and allowed to incubate overnight in a shaking incubator at 37 °C. The overnight cultures were adjusted to an O.D._600nm_ of 0.05 in the modified M9 minimal medium at pH 6.75 (preparation protocol in SI) and 140 μL was added to each well of the 3D printed well plates already containing a single substrate with a CaP:Eu^3+^ coating. At 12 and 24 h the M9 minimal medium was removed prior to measurement and replaced with fresh medium. The bacterial strains used were *P. aeruginosa* (PA01), *K. pneumoniae* (iA565), *E. coli* (DH5α and HVM52). These bacterial species are commonly found in biofilms on orthopedic and catheter implants (Armbruster et al., 2015; Moriarty et al., 2016). Microscopy was performed using a DV Elite microscope (Applied Precision) equipped with a 20x objective on biofilms grown for 48 h on CaP:Eu^3+^ films and stained for 2 h with ECtracer 630 (rebranded to EbbaBiolight 630, EbbaBiotech). The pH values of the biofilm are calculated from a linear spline interpolation of the *S*_*R*_ of the CaP:Eu^3+^ films from pH 4.0 to pH 7.0.

Crystal violet staining was performed on the CaP:Eu^3+^ films after 48 h of incubation at 37 °C by adapting the protocol of (O’Toole, 2011), substrates were rigorously washed with PBS 3 times to remove loosely attached bacteria and moved to a clean well plate. 200 μL of a 0.1% crystal violet in water solution was added and incubated at room temperature for 15 min. The substrates were again rigorously washed 3 times with PBS to remove non-staining crystal violet, 200 μL of 30% acetic acid was subsequently added in order to solubilize the stained crystal violet and absorbance was monitored at 590 nm.

## 3 Results and discussion

**Fig. 1:**
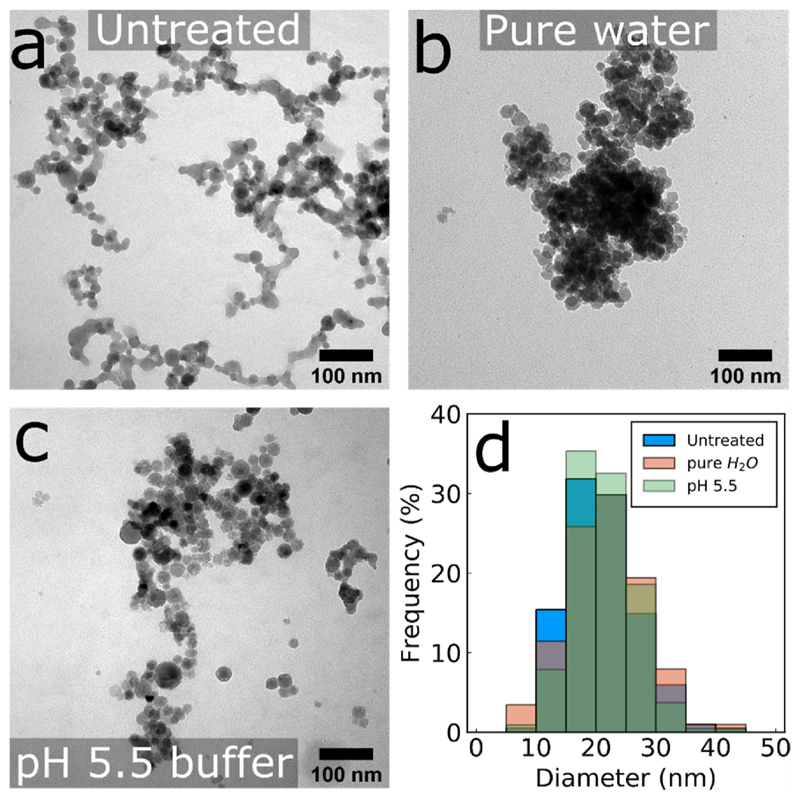
TEM images of (a) CaP:Eu^3+^ nanoparticles made with Ca/P 2.19 ratio and collected downstream and after incubation for 3 h in (b) pure water or (c) pH 5.5 acetic acid buffer. Particle size distributions of the conditions shown in (a)–(c) are shown in (d), counted from a minimum of 200 particles. Average nanoparticles sizes are 20, 21 and 21 nm for untreated, pure water treated and pH 5.5 treated, respectively.

**Fig. 2:**
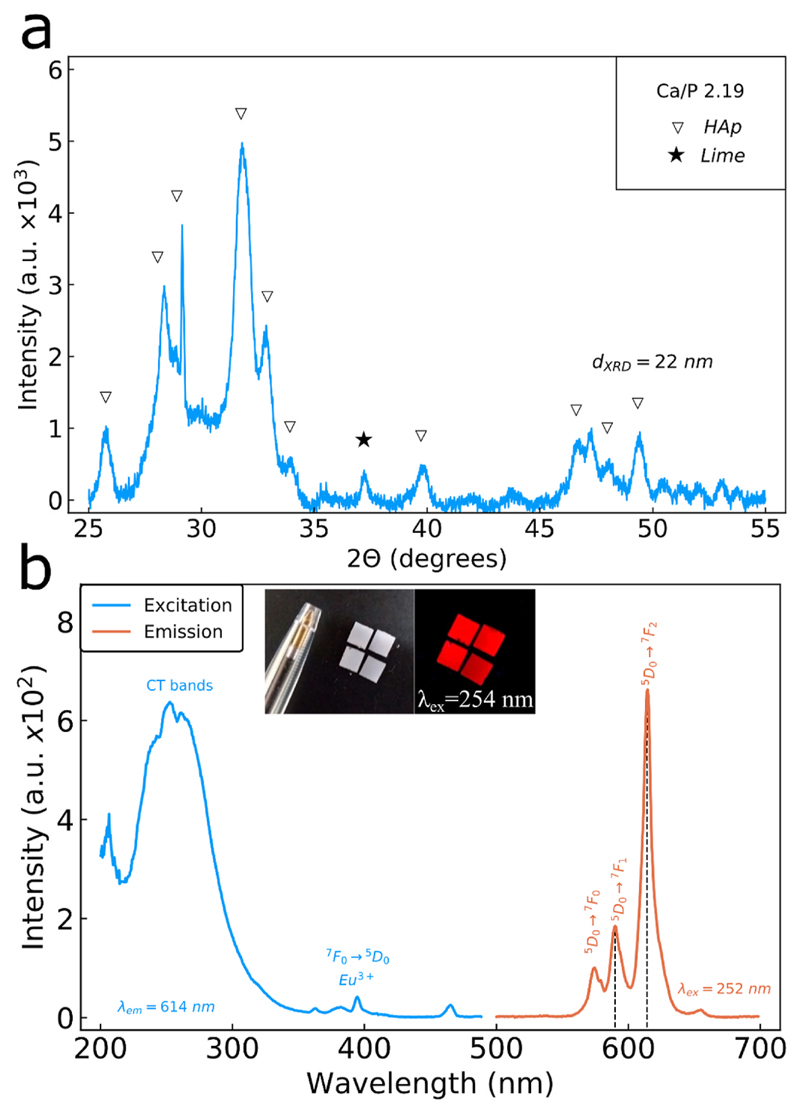
(a) XRD pattern from nanoparticles scraped off the substrates acquired with a 0.1 deg./sec scan speed. Peaks are assigned to the hydroxyapatite (HAp) phase (triangle) and lime phase (filled star). (b) Photoluminescence of the nanoparticle film measured in dry conditions with excitation and emission slits of 3 nm, detector voltage at 900 V, scan speed of 100 nm/min and a total of 10 scans averaged together. The excitation spectrum (blue line) monitored at the emission wavelength of λ = 614 nm showing a characteristic broad excitation of the host lattice at ~250 nm and a sharp excitation peak at λ = 395 nm corresponding to the direct excitation of the Eu^3+^ ions. Emission spectrum (orange line) when excited at λ = 252 nm, demonstrating the characteristic Eu^3+^ ion emission peaks at 570, 592 and 614 nm corresponding to the ^5^D_0_->^7^F_0_, ^5^D_0_->^7^F_1_ and ^5^D_0_->^7^F_2_ transitions, respectively. Insert shows a photograph of the as-deposited nanoparticle films under room light and under UV-light (λ = 254 nm) illumination. (For interpretation of the references to colour in this figure legend, the reader is referred to the Web version of this article.)

The morphology of the CaP:Eu^3+^ nanoparticles (Ca/P 2.19) collected further downstream during their in-situ flame deposition on substrates is evaluated by transmission electron microscopy (TEM) and shown in Fig. 1a, in which an aggregate structure is observed. Upon incubation for 3 h in pure H_2_O (Fig. 1b) or pH 5.5 buffer (Fig. 1c), no significant changes in the morphology or primary particle size distributions (Fig. 1d) are observed. The crystallinity of the deposited nanoparticle films on the substrates is evaluated by powder X-ray diffraction (XRD). Fig. 2a shows the XRD pattern of the nanoparticle film with Ca/P 2.19 that exhibits the characteristic hydroxyapatite crystal phase (COD: 9003549) with a minor calcium oxide phase (96 wt% and 4 wt% respectively, as determined by Rietveld refinement). This low calcium oxide phase in as-prepared crystalline hydroxyapatite nanoparticles at the low pressure drops used here (Pd = 1.8 bar, see Materials and Methods) is seen for the first time in nanoparticles made by flame spray pyrolysis and results from the addition of 2-Ethyhexanoic acid in the precursor solution (SI, Fig. S1) (Ataol et al., 2015; Huber et al., 2005; Nasiri et al., 2016). Furthermore, the crystallinity of the deposited films can be tuned from amorphous to crystalline by varying the nominal Ca/P ratio in the precursor solution (SI, Fig. S2) (Ataol et al., 2015; Huber et al., 2005). No Eu-oxide peaks were detected indicating the incorporation of Eu^3+^ ions into the Ca-phosphate matrix. This Eu^3+^ ion doping renders the as-produced CaP:Eu^3+^ nanoparticles luminescent (Syamchand and Sony, 2015). The in-situ annealed CaP:Eu^3+^ films have a thickness of 14.6 ± 0.7 μm and a porosity of 60 ± 4% (SI, Fig. S3), and with high reproducibility from batch-to-batch (SI, Fig. S4).

The photoluminescence of the as-deposited CaP:Eu^3+^ nanoparticle films is shown in Fig. 2b. The excitation spectrum while monitoring at λ = 614 nm shows the characteristic charge transfer (CT) band at ~250 nm and some minor peaks associated with direct excitation of the Eu^3+^ ions at 395 nm and 470 nm (Ternane et al., 1999). The emission resulting from an excitation at λ = 252 nm shows a characteristic spectrum for Eu^3+^ ions in an asymmetric lattice environment, as determined from the high asymmetry ratio (defined as the ratio of intensities at the wavelengths I_614_/I_592_) (Long et al., 2008). Even though the quantum yield of CaP:Eu^3+^ nanoparticles may be lower than more efficient phosphors such as Y_2_O_3_:Eu^3+^ (Sotiriou et al., 2011) (SI, Fig. S5), the luminescent properties of the as-prepared films (inset in Fig. 2b) allow for their investigation in sensing their chemical envi-ronment in a non-contact manner, as also shown for other rare-earth doped nanophosphors (Henning et al., 2019; Pratsinis et al., 2017).

**Fig. 3:**
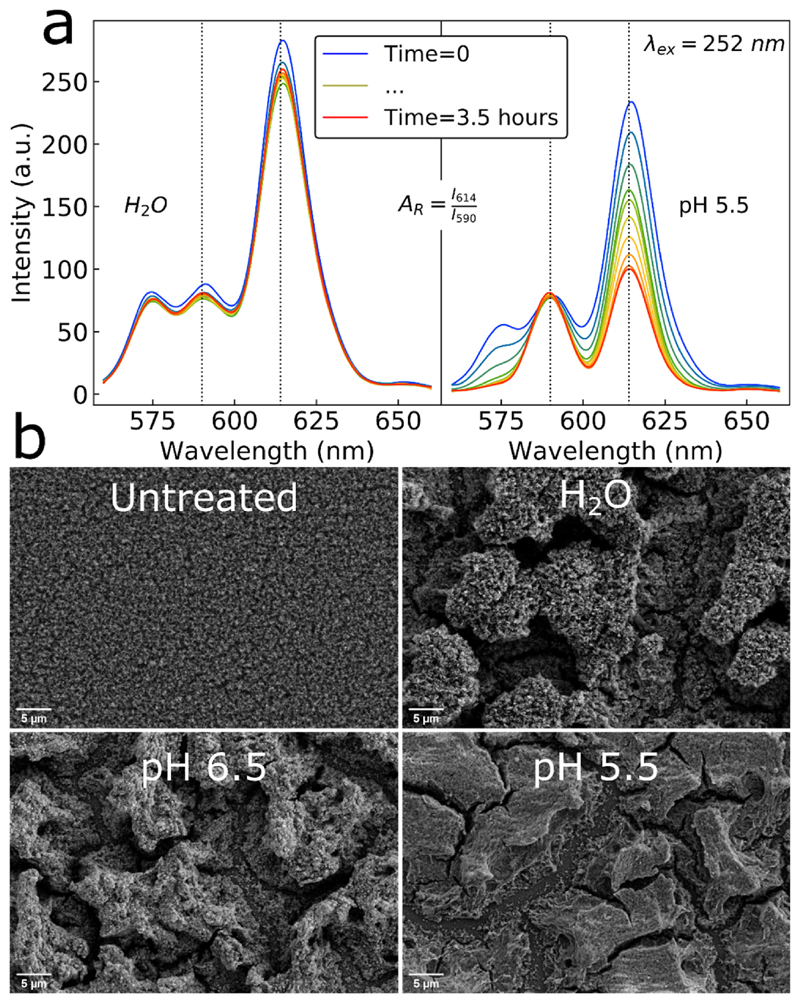
Photoluminescence spectra of CaP:Eu^3+^ coatings in (a) pure water (left panel) and pH 5.5 acetic acid buffer (right panel) demonstrating the clear spectral changes occurring at lower pH, from which the asymmetry ratio (*A*_*R*_) can be established as the ratio of *I*_614_/*I*_590_. (b) SEM images of the nanoparticle films taken after incubation in various pH buffers for 2.5 h.

The in-situ nanoparticle film annealing renders them structurally stable when immersed in solutions (Nasiri et al., 2016; Sotiriou et al., 2013; Tricoli et al., 2008). This stability allows for investigations in various different liquid environments, while simultaneously monitoring their luminescence with a plate reader. Upon the immersion of nanoparticle films with a nominal Ca/P ratio 2.19 in pure water for 3.5 h there is no change in the luminescence spectra (Fig. 3a, left panel). However, upon immersion of identically made films in an acidic (pH 5.5) buffer, the luminescence undergoes a drastic change (Fig. 3a, right panel). More specifically, the luminescence intensity of the peak at 614 nm is quenched, whereas the peak at 590 nm shows minimal change. This allows for a ratiometric readout to be obtained from the asymmetry ratio *A*_*R*_ = $\sum_{\text{n}=610\text{nm}}^{618\text{nm}}{\text{I}}_{\text{n}}/\sum_{\text{n}=586\text{nm}}^{594\text{nm}}I_{n}$. Thus, the sensor response (S_R_) is normalized to the asymmetry ratio (*A*_*R*_) of the dry nanoparticle films prior to incubation and is defined as: $S_{R}=1-A_{R}/A_{R}^{ini}$ where *A*_*R*_ is the measurement after treatment and $A_{R}^{ini}$ is the measurement of the dry film prior to treatment.

Furthermore, the scanning electron microscopy (SEM) images in Fig. 3b show that the CaP:Eu^3+^ nanoparticle films undergo restructuring when immersed in buffer solutions for 2.5 h which becomes more apparent at lower pH, although this could be attributed to the drying of the film prior to SEM analysis. Some nanoparticle film dissolution occurs upon its immersion as measured by calcium ion concentrations (SI, Fig. S6) in supernatants from films treated at pHs of 7 (pure water), 5.5 and 4 (acetate buffers) demonstrating also a clear pH dependent calcium ion release. Nonetheless, the nanoparticle films are still present on the substrates and rather homogeneous upon examining low magnification SEM images of the whole substrates (SI, Fig. S7). Films treated with different acetate buffer concentrations of 0.1 and 0.2 M at pH 5.5 exhibit little difference in the sensor response (SI, Fig. S8). However, a buffer concentration of 0.05 M causes lower sensor response that could be attributed to the mild buffering capacity of the films themselves due to the dissolution of phosphates and carbonates from the films. The CaP: Eu^3+^ nanoparticle films therefore demonstrate a clear pH dependence in their luminescence that may originate from changes in the local elec-tronic environment of the Eu^3+^ ions in the CaP:Eu^3+^ matrix (Lakshminarasimhan and Varadaraju, 2004; Wei et al., 2002).

**Fig. 4:**
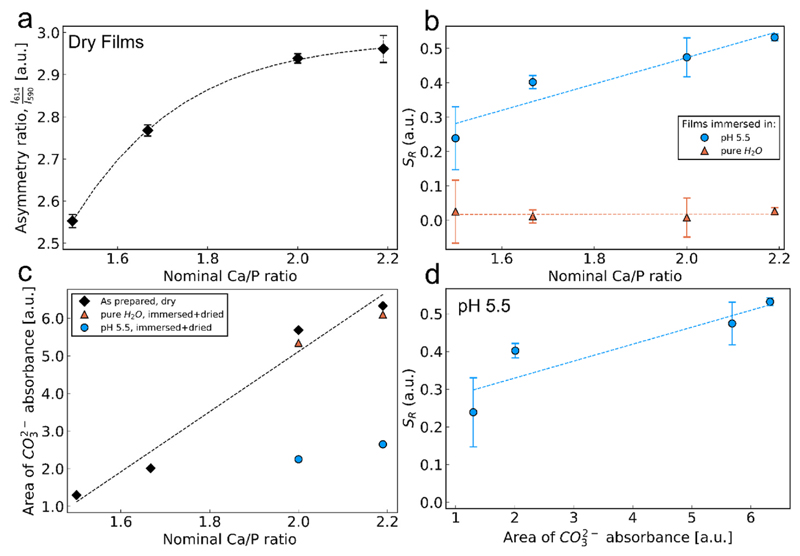
(a) Asymmetry ratios measured dry from the as-deposited films synthesized with various nominal Ca/P ratios. (b) Sensor response *S*_*R*_ as a function of nominal Ca/P ratio of the as-deposited CaP:Eu^3+^ after incubation for 3 h in pure water (red triangles) and pH 5.5 buffer (blue circles). (c) Carbonate content of the films measured with ATR-FTIR spectroscopy of the as-deposited films and the dried 3-h buffer immersion treated chips. (d) *S*_*R*_ as a function of carbonate content of the as-deposited CaP:Eu^3+^ nanoparticle films. (For interpretation of the refer-ences to colour in this figure legend, the reader is referred to the Web version of this article.)

To further study the origin of the sensor response, the effect of the nominal Ca/P ratio in the CaP:Eu^3+^ nanoparticle films is examined in detail. Fig. 4a shows that an increasing Ca/P ratio in the precursor solution yields films with a higher *A*_*R*_ indicating its direct effect on the local structure of Eu^3+^ ions (Wen et al., 2010). Furthermore, upon exposure of all as-deposited films to a pH 5.5 buffer (circles in Fig. 4b) for 3 h, a higher sensor response *S*_*R*_ is obtained for films with higher Ca/P ratio and thus, higher *A*_*R*_. In contrast, all films show a low sensor response *S*_*R*_ when exposed to pure water (triangles, Fig. 4b). The Ca/P ratio also directly affects the carbonate content of the as-deposited films, as determined by Fourier transform infrared (FTIR) spectroscopy, and Fig. 4c shows that higher Ca/P ratios yield films with higher carbonate content as quantified by the $CO_{3}^{2-}$ bands from the FTIR spectra (SI, Fig. S9) (Loher et al., 2005). The carbonate content is not affected when films are exposed for 3 h (and dried) to pure H_2_O (triangles in Fig. 4c), however, when exposed (and dried) to pH 5.5 buffer (circles in Fig. 4c) they show a large decrease in carbonate content.

Upon ex-situ annealing the Ca/P 1.67 nanoparticle films at 900 °C for 3 h the carbonate content is drastically reduced in line with the literature (Lafon et al., 2003) and validated by their FTIR spectra (SI, Fig. S10). The annealed nanoparticle films further show a very low sensor response *S*_*R*_ both in pure H_2_O and pH 5.5 (SI, Fig. S11). This further indicates that the carbonate presence in CaP:Eu^3+^ affects their *S*_*R*_ in acidic environments, even though after annealing the crystallinity of the CaP:Eu^3+^ particles changes drastically (SI, Fig. S12) which might also affect the *S*_*R*_. However, plotting the *S*_*R*_ at pH 5.5 of all nanoparticle films as a function of their carbonate content (Fig. 4d) shows a clear correlation: CaP:Eu^3+^ nanoparticle films with higher carbonate content exhibit higher sensitivity to acidic environments. The presence or absence of carbonate ions in the CaP:Eu^3+^ matrix influences the Eu^3+^ ion’s local electronic environment and thus its asymmetry ratio. These carbonate ions are preferentially dissolved at lower pH modulating the *A*_*R*_ and therefore the *S*_*R*_ of the CaP:Eu^3+^ nanoparticle films in a pH dependent manner. It should be noted that even though surface defects from decreasing particle size might also cause an increase in the *A*_*R*_ (Long et al., 2008), the average primary particle size here for increasing Ca/P ratios remains rather stable as determined by minimal changes of their specific surface area (SI, Fig. S13) and in agreement to the TEM size distribution shown in Fig. 1d. Furthermore, the carbonate content is not easily detected by XRD (Lafon et al., 2003) and therefore there is no significant change in the crystallinity after exposure of the nanoparticles to acidic pH (SI, Fig. S14). It should be noted that the change in *S*_*R*_ resulting from a decrease in carbonated hydroxyapatite of the CaP:Eu^3+^ films content in acidic conditions is irreversible (SI, Fig. S15).

**Fig. 5:**
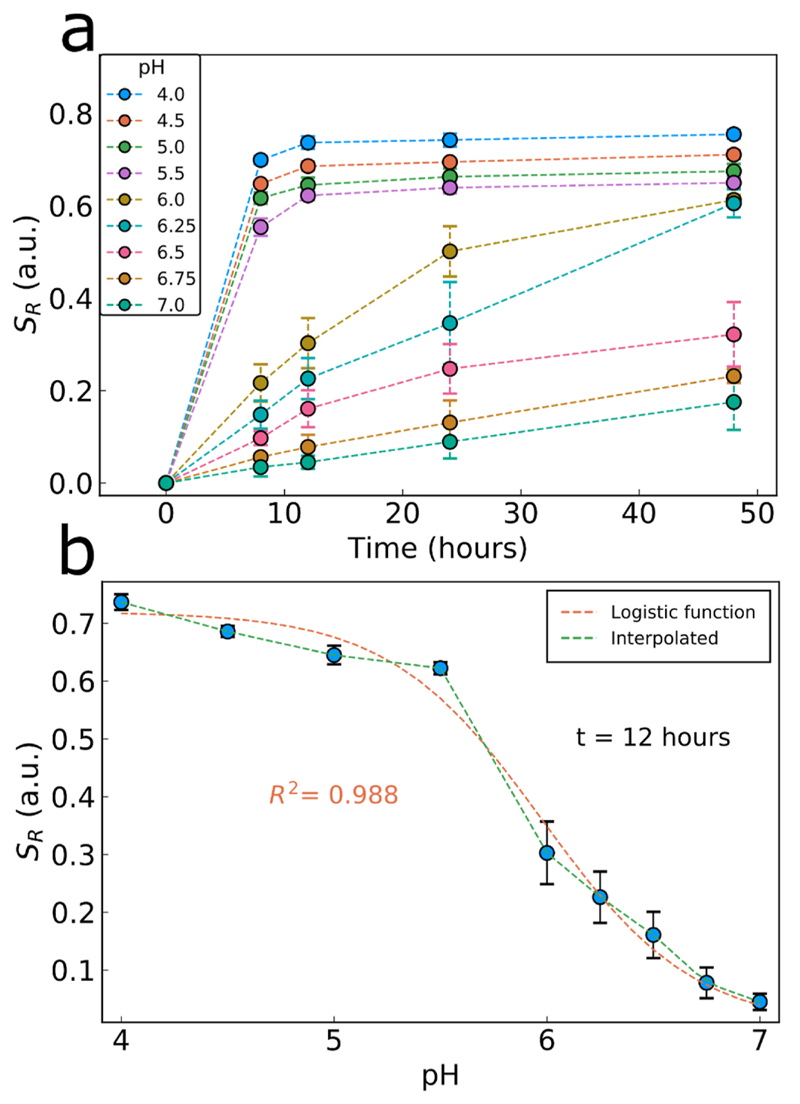
(a) Sensor response *S*_*R*_ in acetic acid adjusted M9 medium across a broad range of pH values. The time-dependent increase in *S*_*R*_ with the tendency towards steady-state. (b) Sensor response at 12 h across the pH range with linear spline interpolation (used for pH calibration curve) and fitted logistic function. Each data points represents the mean of three experiments each performed in triplicate with error bars showing the standard deviation of the three experiments.

The CaP:Eu^3+^ nanoparticles films with the Ca/P ratio of 2.19 which exhibits the highest *S*_*R*_ at pH 5.5 is evaluated further as a ratiometric pH sensor to determine fluorometrically the acidity of its local environment. The *S*_*R*_ of CaP:Eu^3+^ nanoparticle films upon immersion in various pH- adjusted M9 minimal media is monitored over 48 h as shown in Fig. 5a. The films immersed in a low pH medium exhibit a faster and greater change than those incubated in a neutral pH medium and most *S*_*R*_ values appear to reach steady-state over time. From this graph, a calibration curve of *S*_*R*_ as a function of pH can be made for each time point (SI, Fig. S16). For example, the *S*_*R*_ measured after 12 h as a function of pH (Fig. 5b) shows an increase with decreasing pH. The measured *S*_*R*_ gradually increases from pH 7 to pH 4 and upon fitting a logistic function a rather good fit is obtained, however, this fit cannot easily differentiate the low pH values as it treats the region as a plateau. Instead, a linear spline interpolation seems to be more appropriate for this sensor and this is employed further to derive a calibration curve of the sensor response against pH (see SI, Fig. S16 for linear spline fitting at all time-points). Therefore, the potential of the CaP:Eu^3+^ films is demonstrated for their employment as functional pH sensing surfaces for measurements over long periods of time highlighting also their robustness attributed to their all-inorganic nature.

**Fig. 6:**
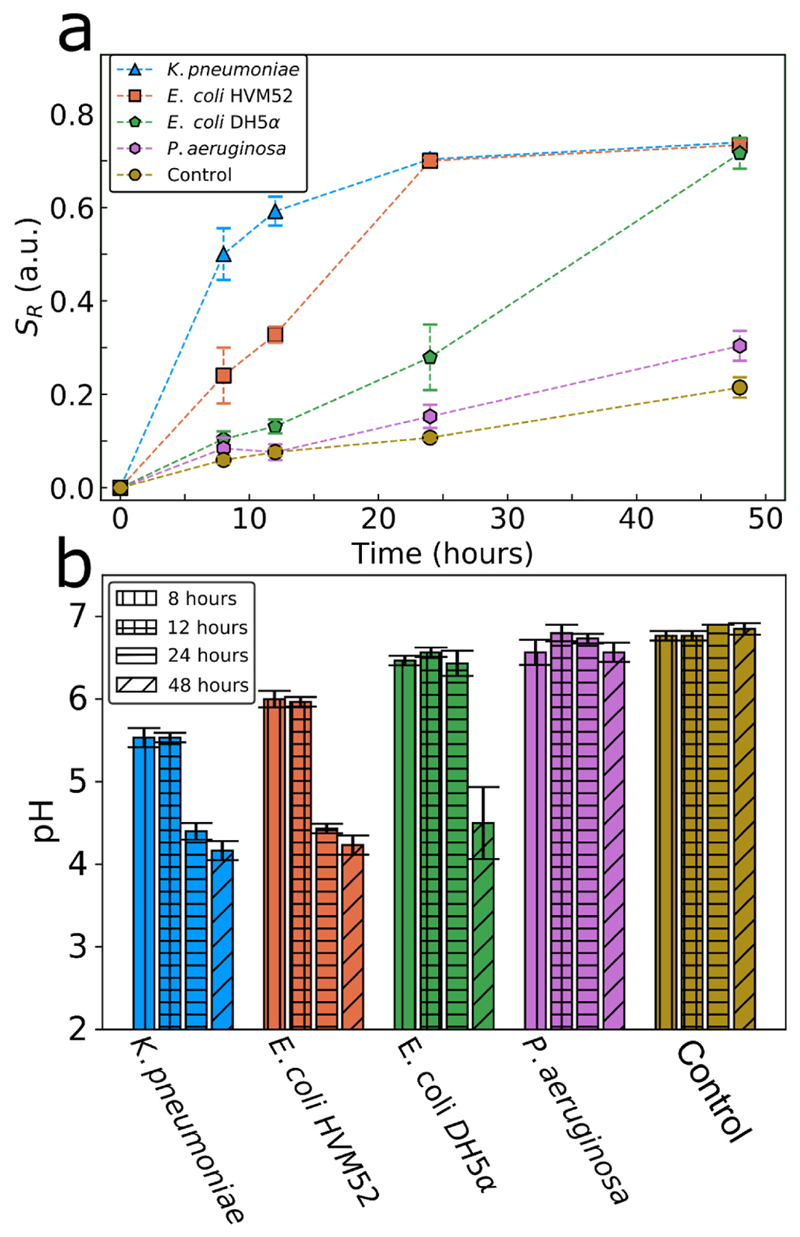
(a) The sensor response *S*_*R*_ values of the pH-sensitive films measured from 4 different bacterial biofilms incubated at 37 °C for 48 h and compared to the values measured in sterile media control. (b) The pH reached by the biofilms over time calculated from the measured sensor response in (a) and the calibration curves at each timepoint (SI, Fig. S16). Each data point represents the mean of three biological replicates (each biological replicate was performed with three technical replicates) and the error bars show the standard deviation of these biological replicates.

The inherent robustness of the as-prepared CaP:Eu^3+^ nanoparticle films allows them to be used in complex environments, such as bacterial cultures and biofilms. Three different clinically relevant gram-negative biofilm-forming bacterial species: *K. pneumoniae, P. aeruginosa* and *E. coli* (strains DH5α and HVM52) are cultured and grown on the CaP: Eu^3+^ films and their luminescence (or *S*_*R*_) is measured over 48 h (Fig. 6a). *K. pneumoniae* exhibits the highest *S*_*R*_ on earlier time-points than the other bacteria indicating a higher acidity followed by *E. coli* with the DH5α stain reaching highly acidic values slower than the HVM52 strain. *P. aeruginosa* shows very little difference in *S*_*R*_ when compared with the control indicating low interfacial acidity.

From the derived *S*_*R*_ and pH calibration curves for each time-point (SI, Fig. S16), the interfacial pH of each biofilm may be determined as shown in Fig. 6b, highlighting that in the conditions used here some bacterial strains are more acidic than others and that this should be considered when studying pH-responsive anti-biofilm strategies. Fig. 6b also shows that the pH in the control conditions does not change over the 48 h studied here. To ensure that the measured *S*_*R*_ indeed originates from the presence of biofilms, the total biofilm biomass was assessed (SI, Fig. S17) using crystal violet staining with a rigorous washing step to remove loosely attached bacteria according to the protocol of (O’Toole, 2011). The presence of biofilm on the nanoparticle film surface after rigorous washing is further assessed with the employment of a fluorescent dye that stains bacterial and biofilm components (SI, Fig. S18).

The lack of correlation between pH and biofilm formation further indicates that the type of organism influences its interfacial biofilm acidity. For instance, in the modified M9 minimal media used here, the biofilm of *E. coli* HVM52 is more capable of producing a low pH interfacial (micro)environment than a biofilm of greater biomass of *P. aeruginosa.* Moreover, the pH values measured here correspond well with those in the literature although the experimental designs are different: an *E. coli* biofilm interfacial pH was measured by Hidalgo et al. (2009) low as pH 4 whereas *P. aeruginosa* biofilms were previously measured by Hunter and Beveridge (2005) to be much less acidic at pH values of no lower than pH 5.9.

## 4 Conclusions

This work demonstrates the fabrication of robust pH responsive all-inorganic luminescent CaP:Eu^3+^ nanoparticle films that were applied to measure the acidity of the solid-biofilm interface. The sensing mechanism was explored in detail and determined to be dependent on carbonate incorporation in the CaP matrix. The pH dependent change in carbonate content induces a change in the local electronic environment of Eu^3+^ ions and thereby modulates the photoluminescence emission. The CaP:Eu^3+^ nanoparticle film sensor was applied for the measurement of the interfacial solid-biofilm pH of three different clinically-relevant gram-negative bacterial species: *K. pneumoniae, P. aeruginosa* and *E. coli* (two strains) in a well plate format and with good agreement with existing literature. The pH sensing nanoparticle films developed here will facilitate the intelligent design of stimuli-responsive surfaces against biofilms.

## Supplementary Material

Supplementary data to this article can be found online at https://doi.org/10.1016/j.bios.2020.112732.

Supplementary File
