# Supplementary material for: Biofilm interfacial acidity evaluation by pH-Responsive luminescent nanoparticle films
Source: Biosens Bioelectron. Author manuscript; Available in PMC 2021 Jan 1. (PMC7116521; doi:10.1016/j.bios.2020.112732)
Supplement: Supplementary File [file EMS108539-supplement-Supplementary_File.pdf]

Supplementary Material

Biofilm Interfacial Acidity Evaluation by pH-Responsive Luminescent Nanoparticle Films

Padryk Merkl[†], *Marie-Stephanie Aschtgen*[†], *Birgitta Henriques-Normark*[†,◊,▲], *and Georgios A. Sotiriou*[*†]

[†]Department of Microbiology, Tumor and Cell Biology, Karolinska Institutet, SE-17177 Stockholm, Sweden.

[◊]Department of Clinical Microbiology, Karolinska University Hospital, SE-171 76 Stockholm, Sweden.

[▲]Lee Kong Chian School of Medicine (LKC) and Singapore Centre on Environmental Life Sciences Engineering (SCELSE), Nanyang Technological University, 639798, Singapore.

*Corresponding author: georgios.sotiriou@ki.se

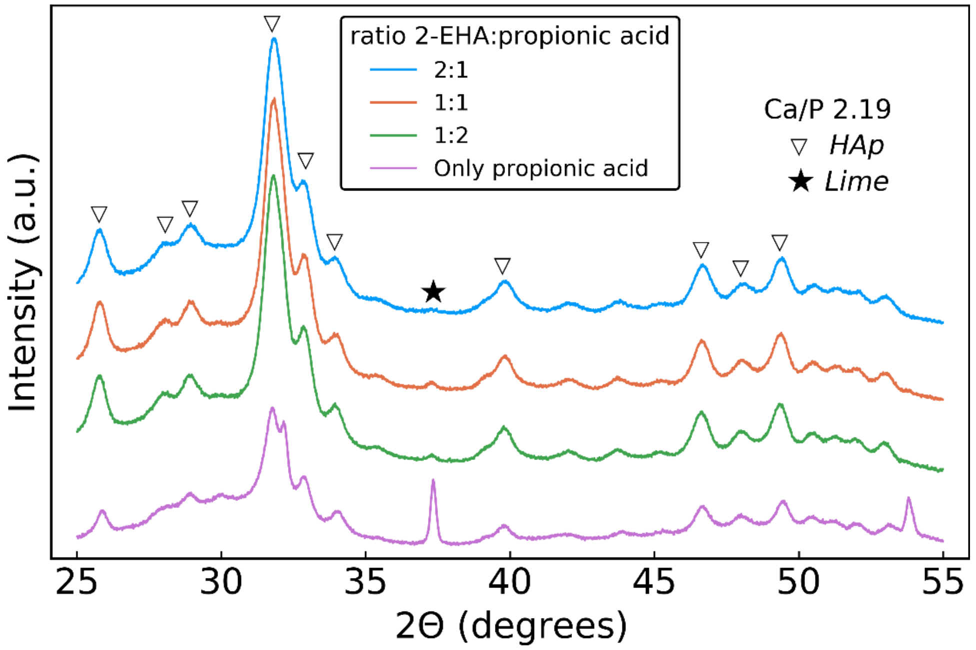

Figure S1. XRD diffractograms of powders collected on filter paper made with changing 2-EHA:propionic acid precursor solvent ratios. The star indicating the CaO phase can be seen to completely disappear at a 2-EHA:propionic acid ratio of 2:1.

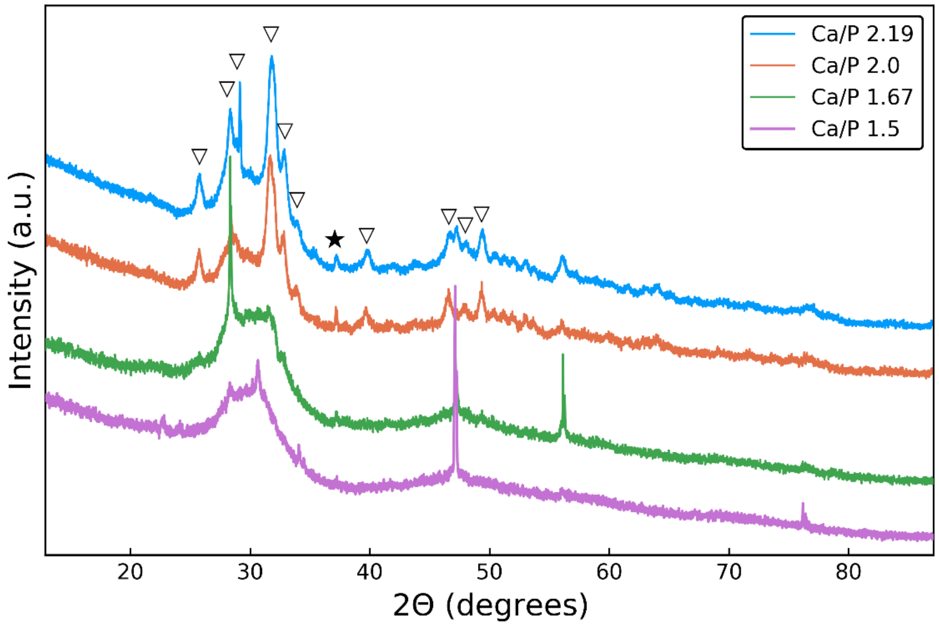

Figure S2. XRD diffractograms of the calcium phosphate films made with a range of Ca/P ratios. Inverted triangles indicate peaks ascribed to the hydroxyapatite phase, the filled star indicates the strongest CaO peak.

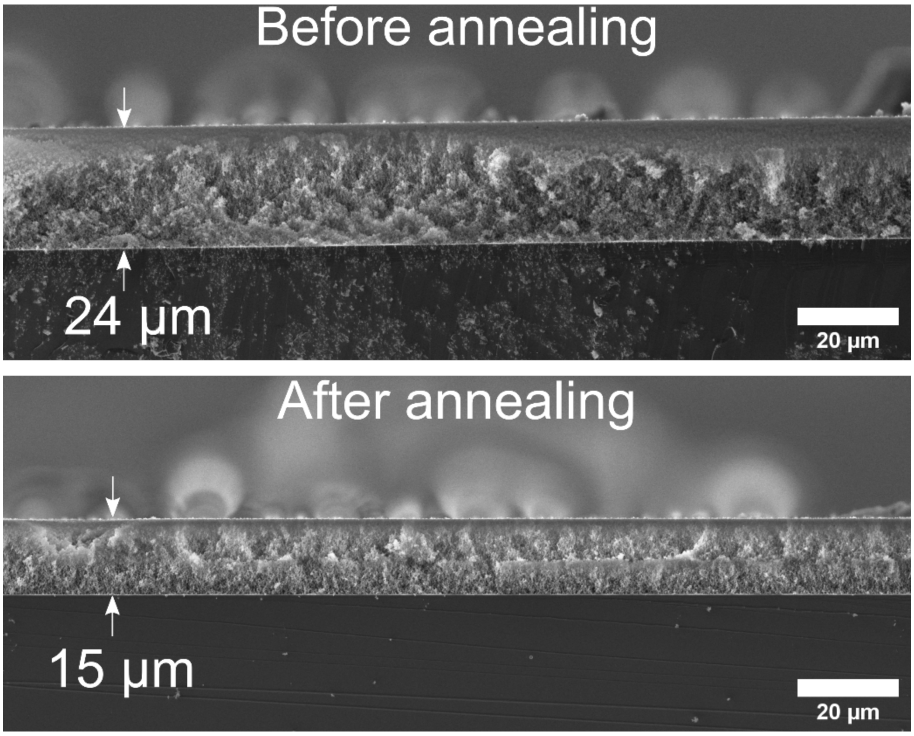

Figure S3. Side-view SEM images of $CaP:Eu^{3+}$ films before and after in-situ annealing demonstrating the compaction of the films. The porosities of the films before and after annealing are 98 % and 60 % respectively.

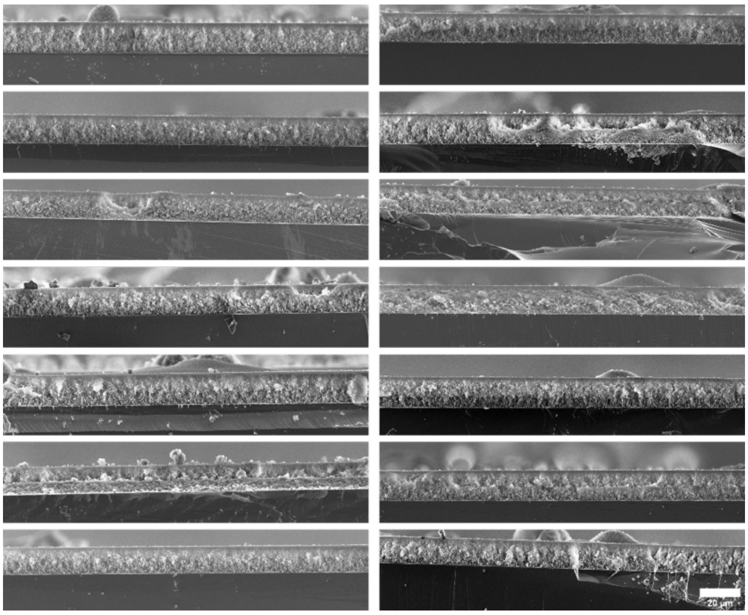

Figure S4. Side-view images of 14 $CaP:Eu^{3+}$ films after in-situ annealing synthesized in 4 different batches demonstrating the reproducible nature of flame spray pyrolysis. These images were used for the calculation of film thickness (14.6 ± 0.7 µm) and porosities (60 ± 4%). Scale bar is the same for all images.

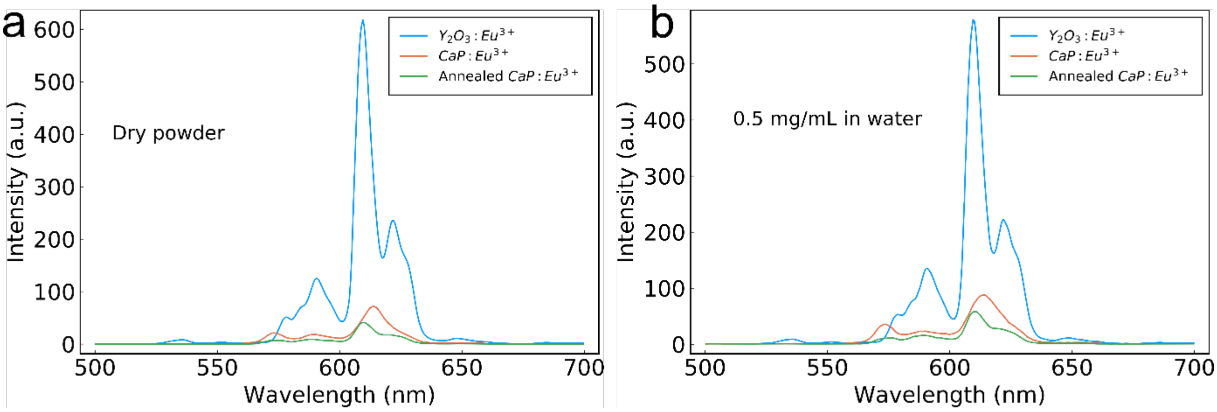

Figure S5. Comparison of the luminescence intensites of the CaP:Eu^{3+} particles used and the well-known nano-phosphor Y_2O_3:Eu^{3+} ((Sotiriou et al., 2011)). Green lines represent CaP:Eu^{3+} particles annealed at 700°C demonstrating lower sensor response as the annealing temperature allows for rearrangement of Eu^{3+} within the lattice to less luminescent sites (Zawisza et al., 2017).

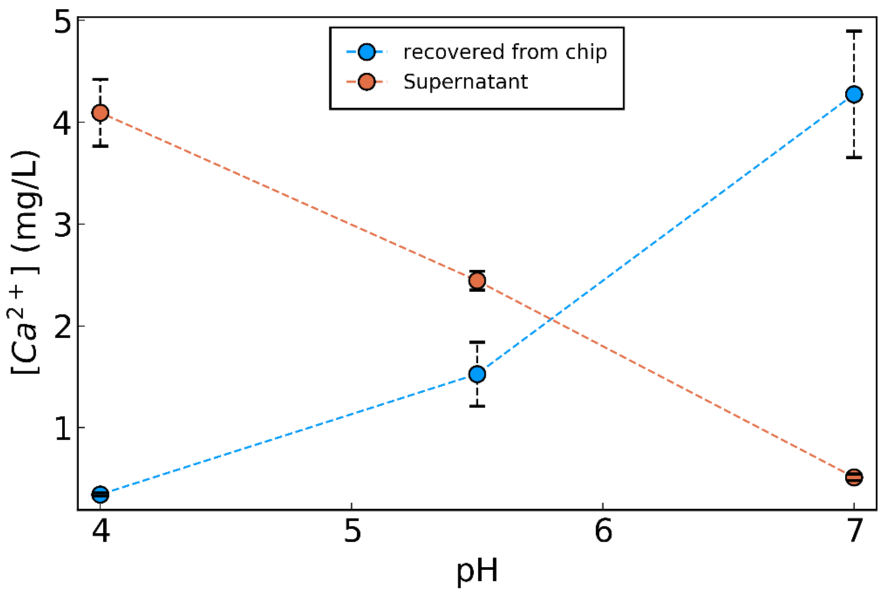

Figure S6. Calcium ion concentrations measured after incubating CaP:Eu^{3+} films for 3 hours in acetate buffers at pHs 4, 5.5 or 7. Measured using a calcium ion selective electrode on the supernatant medium which was incubated with the chips and recovered from the chips by dissolving any remaining CaP:Eu^{3+} in acetic acid.

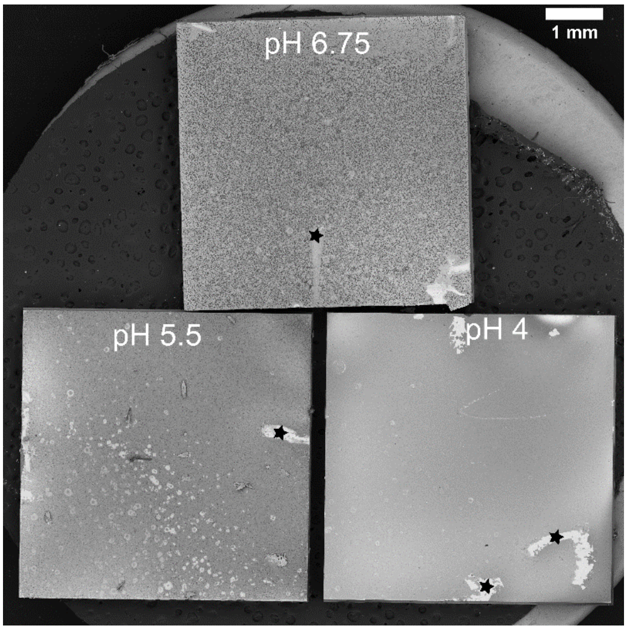

Figure S7. SEM images demonstrating the complete surface coverage of the CaP:Eu^{3+} films after 12 hours of incubation at 37°C in modified M9 minimal medium. Some scratches are visible and marked with black stars due to handling of the films

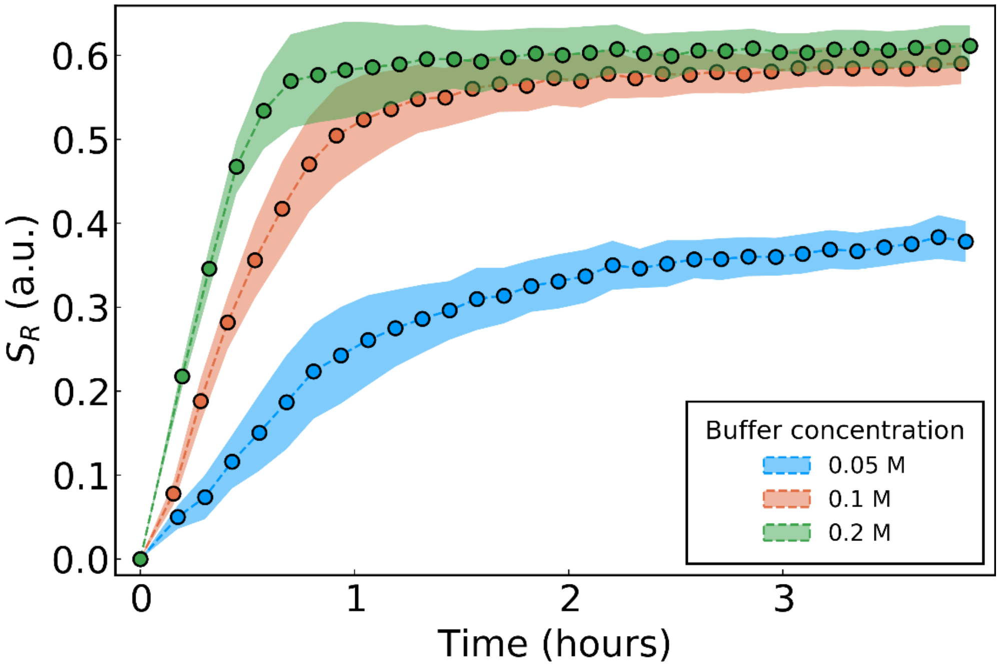

Figure S8. The sensor response from CaP:Eu^{3+} films incubated with different acetate buffer concentrations at pH 5.5.

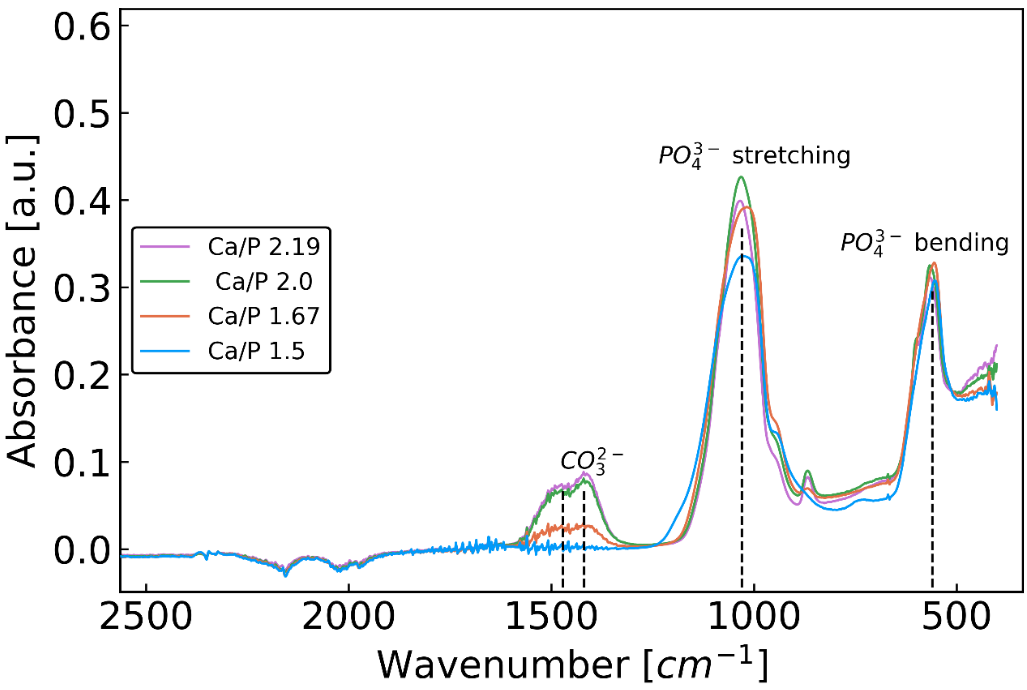

Figure S9. (a) Complete ATR-FTIR spectrum taken from the CaP:Eu^{3+} films synthesized with a range of Ca/P ratios. The carbonate content was determined by integrating the peak at 1400 cm^{-1}.

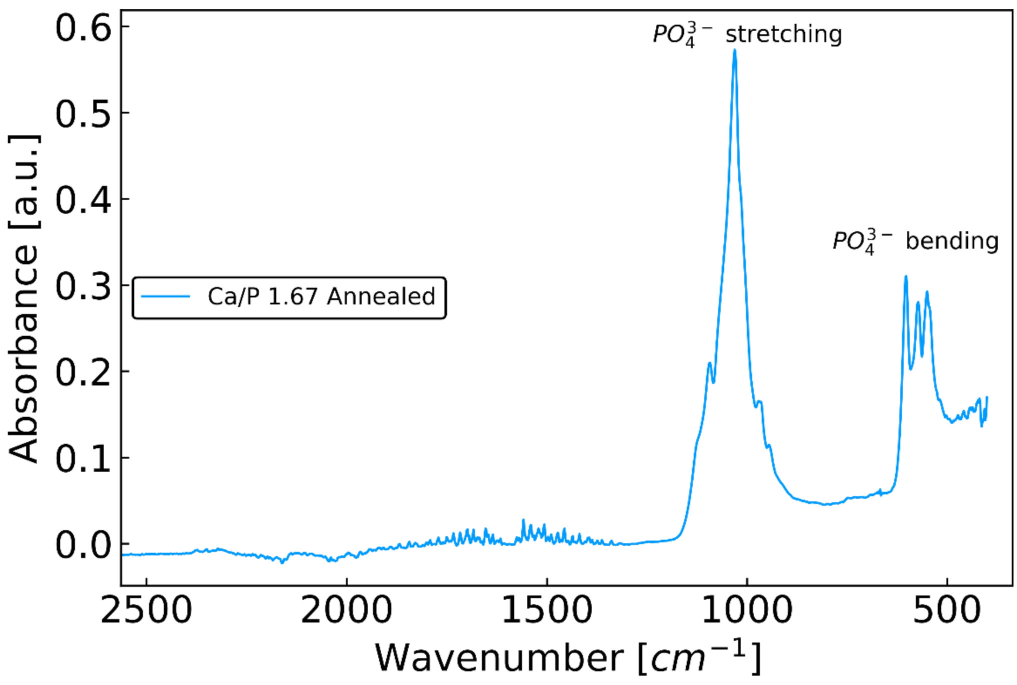

Figure S10. The ATR-FTIR spectrum of a CaP:Eu^{3+} film synthesized with a Ca/P ratio of 1.67 and annealed for 3 hours at 900 °C

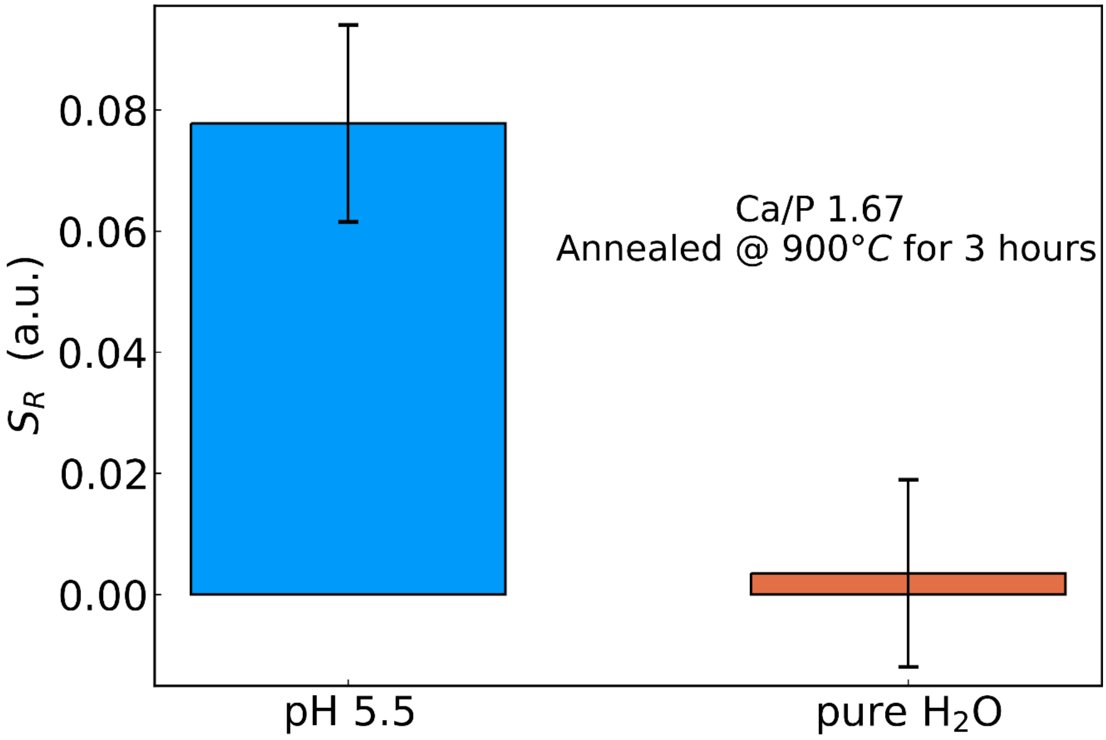

Figure S11. Sensor responses of the coatings annealed for 3 hours at 900°C and subsequently exposed to either pH 5.5 acetic acid buffer or pure H_2O

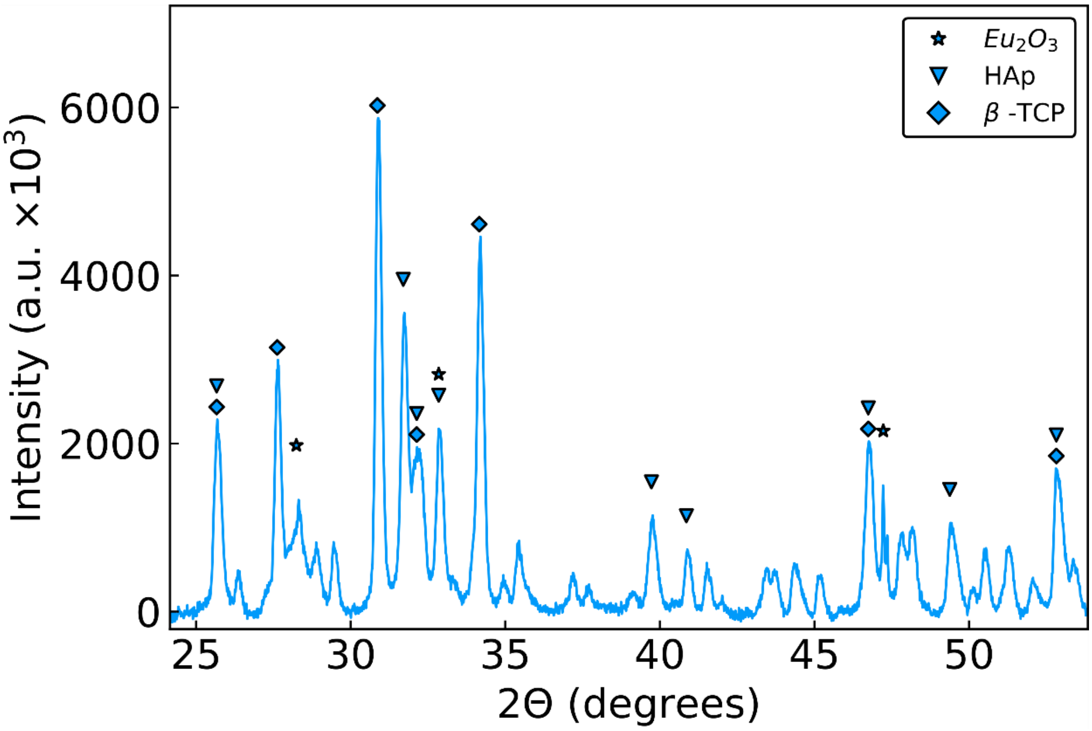

Figure S12. XRD diffractogram of Ca/P 1.667 annealed for 3 hours at 900 °C with relative contents by Rietveld analysis of 60% β-tricalcium phosphate (β-TCP), 37% HAp and 3% Eu_2O_3.

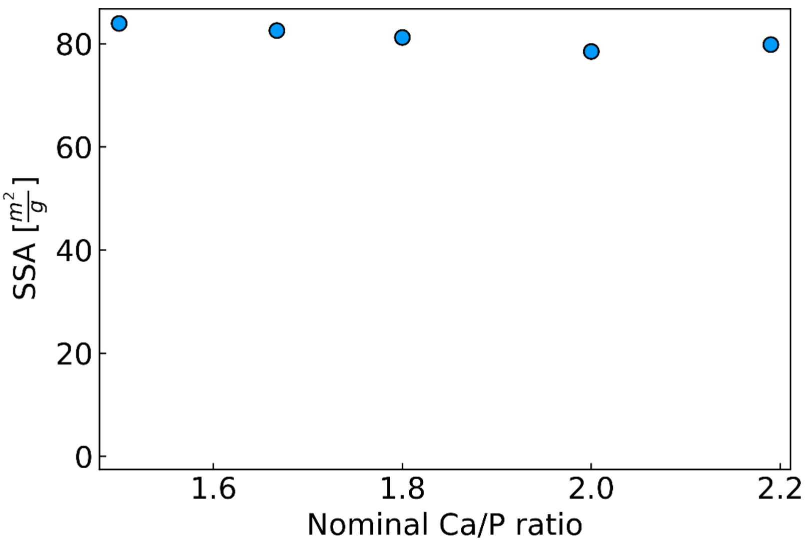

Figure S13. Specific surface area (SSA) calculated by applying BET theory to N_2 adsorption measurements conducted on the nanoparticle powder collected during deposition of CaP:Eu^{3+} films at a range of Ca/P ratios.

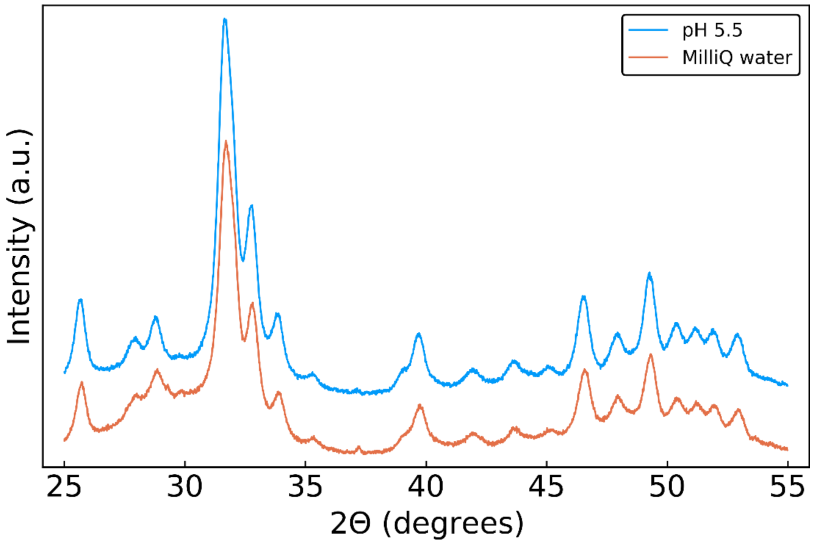

Figure S14. XRD diffractogram taken of nanoparticle powders (Ca/P 2.19) incubated in either pure water or pH 5.5 buffer for 3 hours.

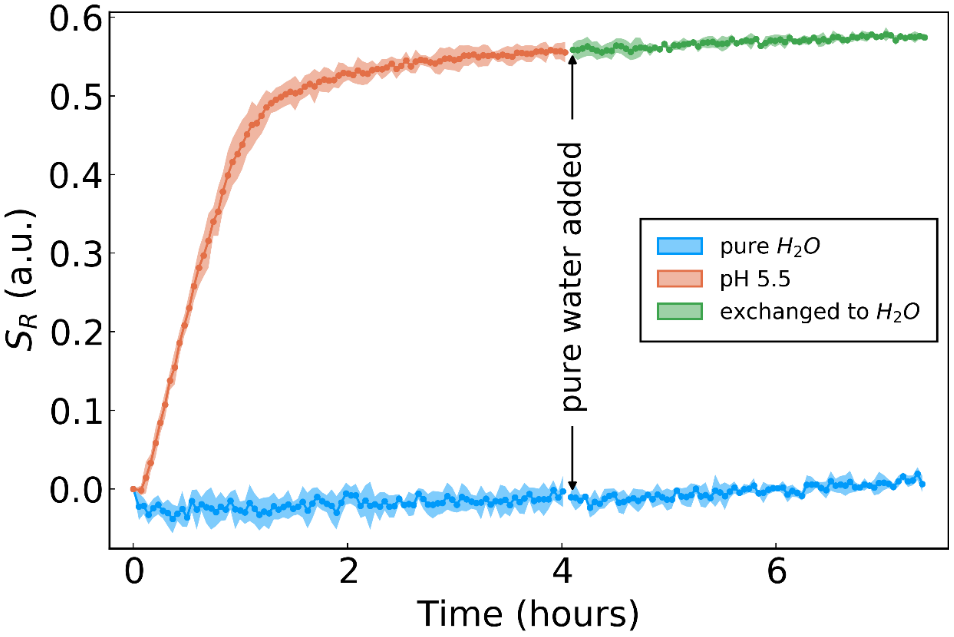

Figure S15. Sensor response of CaP:Eu^{3+} films incubated in either pH 5.5 acetate buffer or pure water (measured to be pH 7). After 4 hours of incubation the mediums were exchanged for fresh pH 7 water.

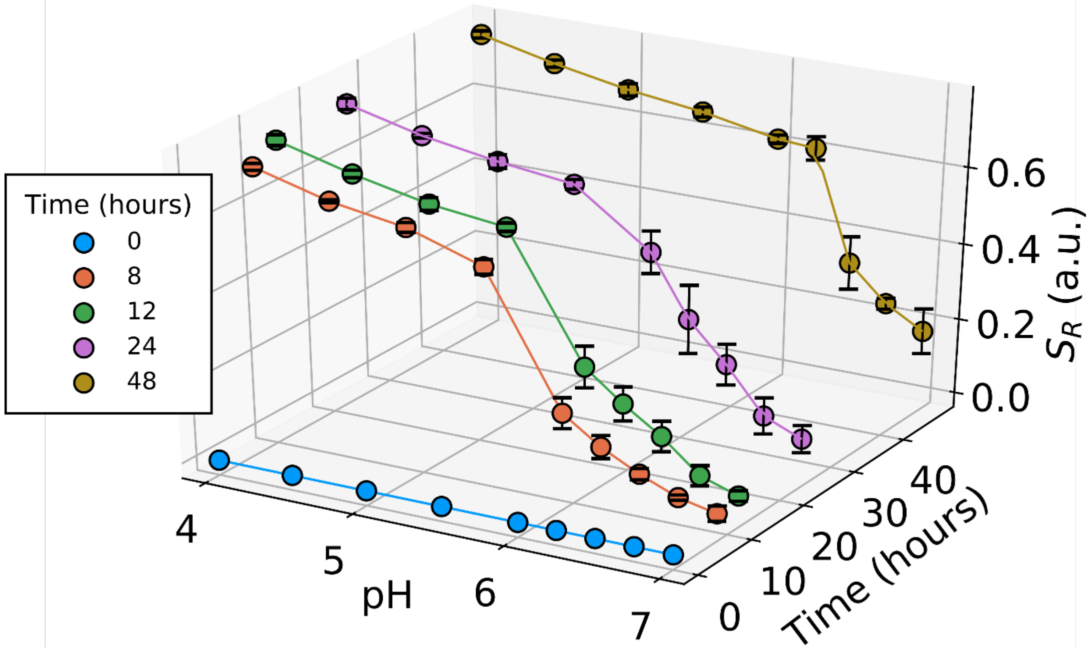

Figure S16. Sensor response measured in acetic acid modified M9 minimal medium incubated at 37°C. With sensor response values interpolated using a linear spline. Each data point corresponds to the mean of at least three separate experiments (each performed in technical triplicate) and error bars show the standard deviation of the separate experiments.

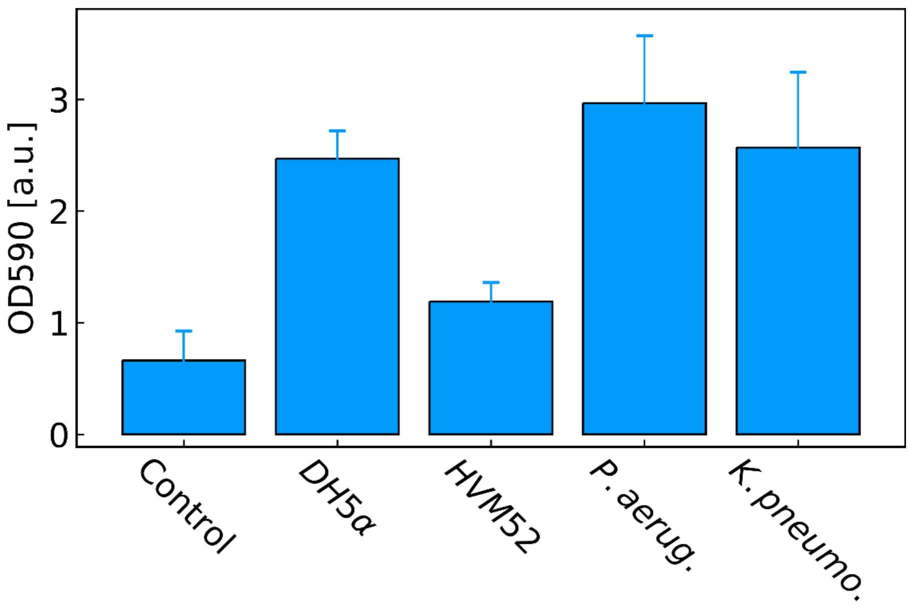

Figure S17. Crystal violet staining performed on biofilms grown on the pH sensitive coatings for 48 hours, showing the difference in biomass formed between the different bacterial strains and species.

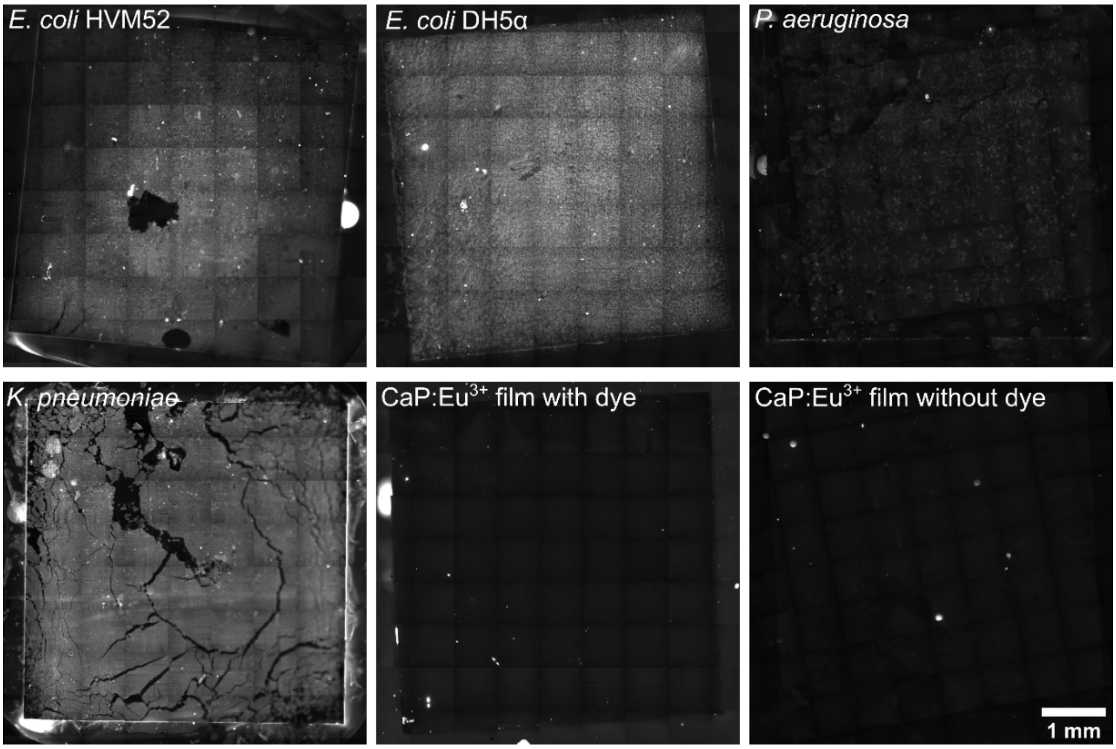

Figure S18. Stitched microscopy images taken with a 20x objective of CaP:Eu^{3+} films after incubation with or without bacteria in modified M9 minimal medium and stained for 2 hours with EbbaBiolight 630 to visualize bacterial biofilms.

Preparation of Modified M9 minimal media:

To 500 mL ultrapure water add:

- 12.8g $Na_2HPO_4.7H_2O$
- 3g KH_2PO_4
- 0.5g NaCl
- 1g NH_4Cl

Autoclave and add sterile filtered:

- 30 mL of $MgSO_4$ 1M stock
- 1 mL of $CaCl_2$ 1M stock
- 1 mL of Thiamine 0.5 M stock
- 133 mL of 30% glucose

Adjust to 1 L with ultrapure sterile filtered water

3D printed well plate technical drawing

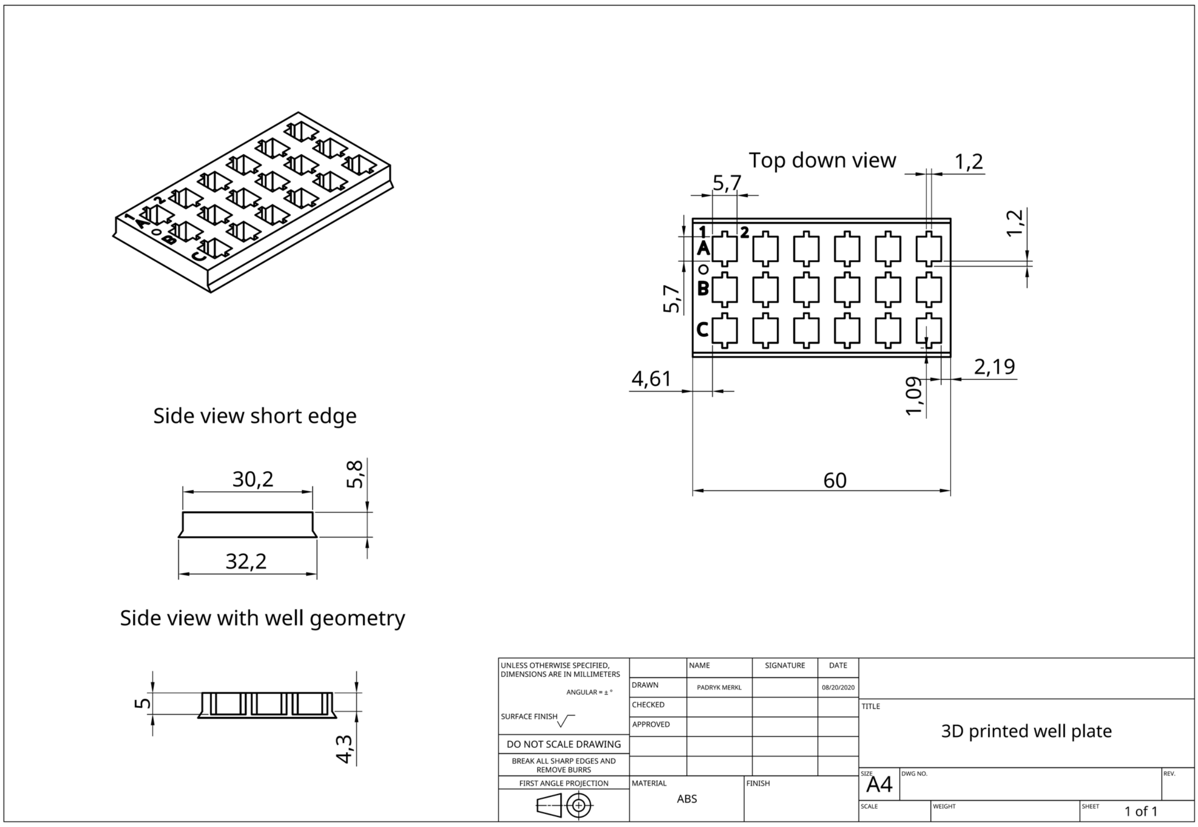

References:

Sotiriou, G.A., Schneider, M., Pratsinis, S.E., 2011. Color-Tunable Nanophosphors by Codoping Flame-Made Y_2O_3 with Tb and Eu. J. Phys. Chem. C 115, 1084–1089. https://doi.org/10.1021/jp106137u

Zawisza, K., Strzep, A., Wiglusz, R.J., 2017. Influence of annealing temperature on the spectroscopic properties of hydroxyapatite analogues doped with Eu^{3+}. New J. Chem. 41, 9990–9999. https://doi.org/10.1039/c7nj01380a
